# Supplementary material for: Patterns of Intron Gain and Loss in Fungi
Source: PLoS Biol. 2004 Nov 30;2(12):e422. doi: 10.1371/journal.pbio.0020422 (PMC532390; doi:10.1371/journal.pbio.0020422)
Supplement: Table S1 — Also available at http://genes.mit.edu/NielsenEtAl/. (4.3 MB ZIP). [file pbio.0020422.st001.zip › NielsenEtAl/html/696.html]

AN1524.1.NCU00963.1.MG10834.1.FG00417.1


```
 CLUSTAL W (1.82) Multiple Sequence Alignments - Introns Inserted


Sequence 1: MG10834.1	325 aa
Sequence 2: FG00417.1	381 aa
Sequence 3: NCU00963.1	342 aa
Sequence 4: AN1524.1	334 aa
Alignment Length: 386 aa
Number Identitical Residues: 168 aa
Alignment Score (without introns) 8068


MG10834.1 	------~--------~-------------~---------------MDSTTEVPKTCKVVT
NCU00963.1	------~--------~-------------~----------------MEAQQVPKTCKVIL
FG00417.1 	MAKLYT0INPDFSKV1RLMAKIMWGWLLS0IKPCFRSLSLNLTTFAMASSTVPKTCKVIT
AN1524.1  	------~--------~-------------~--------------MATPSEPAPTNCKVML
          	                                                :  .*..***: 

MG10834.1 	AETVAKSLLEEVRS-----QKGTDSKPYLAAFLANDDPAALQYAQWSKKTCEEN2GFEFD
NCU00963.1	SDTIAKKLLSEVRETLAKIEGPNASKPTLAAFLATDDPAALQYAEWSKRTCEEN2GFNFD
FG00417.1 	AETIAKGFLGEVKDTLAKIQGSNPCTPTLAAFLANGDPAAVKYAEWSKKTCEEN2GFNFD
AN1524.1  	SKHVANGLLAEVSEG----IKTLEKPPHLVGFLANNDPAALMYAQWTEKTCHEN2GFRYS
          	:. :*: :* ** .            * *..***..****: **:*:::**.** **.:.

MG10834.1 	LRQVGKDALEEDIIAANEDAKVDGIIVYYPIFPGNPTHDKYIQETVALEKDVEGLCHKHL
NCU00963.1	LRKVDKENLEEGIIAANNDDKVDGILVYYPIWVGNHSQDKYIKETVALSKDVEGLCHTHL
FG00417.1 	LRTVDKELLEEEIMKANEDEDVDGMIVYYPIFPNNPSHDKYIQESVDLGKDVEGLRHQHI
AN1524.1  	LREVHRDNLEEAILAANVDPDVDGIIVYYPIF--NNRQDQYLQQIVDVSKDVEGLSHRYI
          	** * :: *** *: ** * .***::*****:  *  :*:*::: * : ****** * ::

MG10834.1 	YNMYHNVRFLDPPTNSKKSILPCTPLAVVKILEHLQIYNSILPYGNRLYGKTVTVINRSE
NCU00963.1	FNMYHNVRFLDPPTNLKKSILPCTPLAIVKTLEYLQIYNPILAYGNRLFGKTITVINRSE
FG00417.1 	HNMYHNIRFIDPPENHKKSILPCTPLAVVKILEYLQIYNPILASGNRLYGKTITVINRSE
AN1524.1  	FNMYQNIRFLDPETKRQKCILPCTPLAIVKILEHLNIYNTVLPYGNRLHGHTICVVNRSE
          	.***:*:**:**  : :*.********:** **:*:***.:*. ****.*:*: *:****

MG10834.1 	VNGRPLAALLANDGAKVYSVDVTGVQLFTRGQGIRQPRHQVLDKEDWGLKECIPLSDVII
NCU00963.1	VNGRPLAALLANDGATVYSVDITGVQVFTRGEGIRQLRHQVHDKEGWELKDVLPLSDVVI
FG00417.1 	VNGRPLAALLANDGATVYSVDITGVQVFTRGEGIKKARHLVQDKEGWKLEDCLPLSDVVI
AN1524.1  	VVGRPLAALLANDGACVYSVDITGIQKFYRGTGLKLGRHEVEDVEGKELKDIVPLCDTVI
          	* ************* *****:**:* * ** *::  ** * * *.  *:: :**.*.:*

MG10834.1 	GGVPVAEFKIPVELIREGAVCINFSSFR~NFDGPAVKEKASIYVPSVGKVTIAVLLRNLV
NCU00963.1	GGVPTEKFKVPTELLRDGAVCINFSSFK~NFDGPAVKEKASIYVPSIGKVTIAVLLRNLV
FG00417.1 	GGVPVESFKVPTELIREGAVCINFSSYR~NFDGPAIKEKASIYVPSVGKVTIAILLRNLV
AN1524.1  	SGVPGDKYKFDTSLLRDGAVCINFSSEK0NFG-PEVKQKASIFVPSIGKVTIVVLLRNLL
          	.***  .:*. ..*:*:********* : **. * :*:****:***:*****.:*****:

MG10834.1 	0RLIANR----------NPGIEAAAAKS----
NCU00963.1	0RLIANRPRPEGSAPEDPKDAKARSEAFIEG-
FG00417.1 	0RLIANRP----SKDESQKNVEARAEAFADD-
AN1524.1  	0RLIQNKR----MDDIKPAAATERPGTLEAAS
          	 *** *:         .       .      :
```
